# Supplementary material for: Mechanism Repositioning Based on Integrative Pharmacology: Anti-Inflammatory Effect of Safflower in Myocardial Ischemia–Reperfusion Injury
Source: Int J Mol Sci. 2023 Mar 10;24(6):5313. doi: 10.3390/ijms24065313 (PMC10048972; doi:10.3390/ijms24065313)
Supplement: Supplementary file 1 [file ijms-24-05313-s001.zip › Supplementary File S1.pdf]

Tabel S1. Identifying the components in ESI$^+$ mode

NO.	Component name	Formula	Theoretical (m/z)	Observed (m/z)	Mass error (ppm)	tR (min)	Response	Adducts	Ion fragment
1	Vanillic acid	$C_8H_8O_4$	168.0423	169.0754	-3.9	0.57	14781	+H	95.0457[M+H-CH_3O-CHO_2]
2	Serotobenine	$C_{20}H_{18}N_2O_4$	350.1267	351.1358	-1.7	1.18	5902	+H	337.1384[M+H-NH]/166.0710[M+H-$C_9H_9N_2O$]/156.0726[M+H-$C_9H_8O_3$]
3	N-(m-coumaroyl) tryptamine	$C_{19}H_{18}N_2O_2$	306.1368	329.1250	-3.3	1.33	6529	+Na	146.0594[M+H-$C_{10}H_8N$-OH]/130.0498[M+H-$C_{10}H_9N_2$-OH]/121.0645[M+H-$C_{11}H_{10}ON_2$]/
4	Roseoside	$C_{19}H_{30}O_8$	386.1941	409.1813	-4.8	1.57	40101	+Na	175.1178[M+H-OGlc-OH-CH_3]/144.1119[M+H-OGlc-C_2H_2O-OH]
5	Tracheloside	$C_{27}H_{34}O_{12}$	550.2050	551.2148	4.6	1.62	6049	+H	451.1397[M+H-$C_2H_6O_2$-2OH]/322.0539[M+H-2OH-CH_3O]/284.0983[M+H-$C_{14}H_{16}O_5$]
6	Quercetin-3-O-β-D-glucoside	$C_{21}H_{20}O_{12}$	464.0955	465.1025	-0.4	2.12	509532	+H	296.0987[M+H-$C_6O_3H_4$-CH_3O-OH]/147.0394[[M+H-OGlc-$C_6H_4O_3$-OH]
7	D-Phenylalanine	$C_9H_{11}NO_2$	165.0790	166.0856	-4	2.23	388345	+H	120.0805[M-COOH]/91.0539[M+H-$C_2H_4NO_2$]
8	6-hydroxykaempferol-6,7-O-β-D-glucoside	$C_{27}H_{30}O_{17}$	626.1483	627.1547	-1.5	2.26	32122	+H	465.1028[M+H-Rha]/451.1413[M+H-$C_9H_6O_3$-OH]
9	Chlorogenic acid	$C_{16}H_{18}O_9$	354.0951	377.1599	-1.2	3.34	76853	+Na	201.0458[M+H-$C_8H_7O_2$-OH]/137.0595[M+H-$C_7H_8O_7$]
10	N-[2-(5-Hydroxy-1H-indol-3-yl) ethyl]-p-coumaramide	$C_{19}H_{18}N_2O_3$	322.1317	323.1300	2.3	3.37	5239	+H	156.0765[M+H-$C_9H_7O_2$]/147.0438[M+Na-$C_{10}H_{11}ON_2$]/119.4924[M+H-$C_{11}H_{11}ON_2$]

11	Flavanonol	$C_{15}H_{12}O_3$	240.0786	241.1117	-2.8	3.61	5943	+H	167.0723[M+H-C_6H_4]/147.0432[M+H-C_6H_4-OH]
12	1-(O-2,4,6-trimethoxyphenyl)-6-(O-galloyl)-β-D-glucopyranoside	$C_{22}H_{26}O_{13}$	498.1373	499.0982	-4.3	4.18	6287	+H	451.1129[M+H-2OH-CH_3]/433.1125[M+H-3OH-CH_3]/267.0958[M+H-$C_9H_{11}O_4$-3OH]
13	Saffloquinoside B	$C_{33}H_{36}O_{17}$	704.1953	705.1819	-3	4.41	20533	+H	461.1065[M+H-Rha-C_6H_5O]/299.0544[M+H-Glc-Rha]/287.0544[M+H-Glc-Rha-C_6H_5O-OH]
14	N-feruloyltryptamine	$C_{20}H_{20}N_2O_3$	336.1474	337.1818	1.7	4.52	9541	+H	188.0702[M+H-C_8H_7N-OH]/147.0437[M+H-$C_{10}H_{10}N_2$]
15	Saffloquinoside C	$C_{27}H_{32}O_{17}$	628.1640	629.1696	-2.5	4.56	178326	+H	467.1180[M+H-Glc]/367.0997[M+H-Glc-C_7H_6O]
16	6-Hydroxykaempferol-3,6,7-triglucoside	$C_{33}H_{40}O_{22}$	788.2011	789.2061	-2.9	5.09	692995	+H	627.1546[M+H-Glc]/465.1026[M+H-2Glc]/303.0495[M+H-3Glc]
17	Hydroxysafflower yellow A	$C_{27}H_{32}O_{16}$	612.1690	613.1753	-1.7	5.24	4210682	+H	451.1235[M+H-Glc]/433.1127[M+H-Glc-OH]
18	Safflower yellow B	$C_{48}H_{54}O_{27}$	1062.2853	1063.2894	-2.9	5.86	60993	+H	737.1863[M+H-Glc-$C_9H_7O_2$-OH]/575.1391[M+H-2Glc-$C_9H_7O_2$-OH]
19	Lirioresional A	$C_{21}H_{24}O_8$	404.1471	427.1627	1.4	5.97	29101	+Na	213.1220[M+H-$C_{10}H_{10}O_5$]/167.6272[M+H-$C_{11}H_{11}O_5$]
20	Scutellarin	$C_{21}H_{18}O_{12}$	462.0798	463.0870	-0.1	6.53	27192	+H	287.0546[M+H-$C_6H_9O_6$]/271.0585[M+H-$C_6H_9O_7$]
21	Naringenin	$C_{15}H_{12}O_5$	272.0685	273.0752	-1.9	6.61	24611	+H	179.0332[M+H-C_6H_5O]

22	(E)-Ferulic acid	$C_{10}H_{10}O_4$	194.0579	195.0647	-2.6	7.11	11053	+H	177.0542[M+H-H_2O]/149.5932[M+H-CHO_2]
23	Eriodictyol	$C_{15}H_{12}O_6$	288.0634	289.0705	-0.4	7.31	306217	+H	147.0438[M+H-OH-$C_6H_4O_3$]/119.0488[M+H-OH-$C_7H_4O_4$]
24	(2S)-4',5-dihydroxyl-6,7-di-O-β-D-giucopyanosyl flavanone	$C_{27}H_{32}O_{16}$	612.1690	635.1579	-0.5	7.32	206914	+Na	595.1659[M-OH]/433.1129[M+H-Glc]/289.0705[M+H-2Glc]/147.0438[M+H-2Glc-$C_8H_5O_2$]
25	Myricetin	$C_{15}H_{10}O_8$	318.0376	319.0445	-1.2	7.77	41466	+H	193.4080[M+H-$C_6H_5O_3$]/152.0030[M-H-$C_8H_6O_4$]
26	6-Hydroxykaempferol 3-Rutinoside-6-glucoside	$C_{33}H_{40}O_{21}$	772.2062	773.2117	-2.3	8.24	1113254	+H	627.1546[M+H-Rha]/465.1025[M+H-Rha-Glc]/303.0494[M+H-Rha-2Glc]
27	6-hydroxykaempferol 3,6-diglucoside	$C_{27}H_{30}O_{17}$	626.1483	627.1543	-2	8.29	1492252	+H	465.4025[M+H-Glc]/303.0494[M+H-2Glc]
28	6-hydroxyapigenin-6-O-β-D-glucoside-7-O-β-D-glucuronide	$C_{27}H_{28}O_{17}$	624.1327	625.1389	-1.7	8.4	727220	+H	463.0868[M+H-Glc]/287.0544[M+H-Glc-$C_6H_{12}O_6$]
29	Saffomin C	$C_{30}H_{30}O_{15}$	630.1585	631.1636	-3.5	8.42	27087	+H	564.1703[M+H-4OH]/402.1175[M+H-OGlc-3OH]/287.0544[M+H-OGlc-$C_9H_8O_3$]
30	Safflower yellow A	$C_{27}H_{30}O_{16}$	610.1534	611.1596	-1.8	8.47	166592	+H	449.1075[M+H-Glc]/433.1122[M+H-OGlc]/247.0229[M+H-OGlc-$C_9H_7O_2$]

31	Scopoletin	$C_{10}H_8O_4$	192.0423	193.0489	-3.4	8.5	67667	+H	178.0253[M+H-CH_3]
32	Saffloquinoside A	$C_{27}H_{30}O_{15}$	594.1585	617.1463	-2.3	8.8	26961	+Na	433.1115[M+H-Glc]/287.0541[M+H-Glc-$C_6H_7O_4$]
33	Rutin	$C_{27}H_{30}O_{16}$	610.1534	611.1604	-0.4	9.35	732202	+H	465.1030[M+H-Rha]/303.0498[M+H-Glc-Rha]
34	Yellow pigment A	$C_{26}H_{28}O_{16}$	596.1377	597.1703	-2	9.35	11390	+H	303.0498[M+H-OGlc-2OH-C_6H_5O]/169.0125[M+H-2Glc-C_8H_7O]
35	6-Hydroxykaempferol	$C_{15}H_{10}O_7$	302.0427	303.0497	-0.8	9.47	1375662	+H	287.0547[M-OH]/169.0127[M+H-$C_8H_5O_2$]
36	6-hydroxykaempferol-3-O-β-D-glucoside	$C_{21}H_{20}O_{12}$	464.0955	465.1030	0.6	9.47	776885	+H	449.1077[M-OH]/303.0497[M+H-Glc]/287.0547[M+H-OGlc]/169.0127[M+H-OGlc-C_8H_5O]
37	Kaempferol-3-O-sophoroside	$C_{27}H_{30}O_{16}$	610.1534	633.1414	-2	9.49	93981	+Na	303.0497[M+H-Glc-$C_6H_7O_3$]/287.0547[M+H-2Glc]
38	N,N'-[2,2'-(5,5-dihydroxy-4,4'-bi-1H-indol-3,3'-yl)diethyl]-diferulamide	$C_{38}H_{32}N_4O_6$	640.2322	663.2187	-3.6	10.1	55258	+Na	477.1937[M+H-$C_9H_7O_2$-OH]/238.0700[M+H-C_6H_5O-$C_9H_7O_2$-$C_{10}H_8ON$]
39	6-hydroxykaempferol-7-O-glucoside	$C_{21}H_{20}O_{12}$	464.0955	465.1032	1	10.48	13422	+H	287.0545[M+H-Glc]
40	Quercetin	$C_{15}H_{10}O_7$	302.0427	303.0497	-0.6	10.66	233661	+H	285.0398[M+H-H_2O]/153.0176[M+H-$C_8H_5O_3$]
41	Carthamone	$C_{21}H_{20}O_{11}$	448.1006	449.1077	-0.3	10.76	24840	+H	169.0125[M+H-Glc-C_8H_7O]

42	Syringin	$C_{17}H_{24}O_9$	372.1420	395.1318	1.3	11.59	24058	+Na	191.0674[M+H-Glc-OH]/137.0595[M+H-OGlc-C_3H_5O]
43	kaempferol-3-O-β-rutinoside	$C_{27}H_{30}O_{15}$	594.1585	595.1656	-0.3	12.17	1177687	+H	287.0548[M+H-2Glc]/153.0178[M+H-2Glc-C_8H_5O]
44	Kaempferol	$C_{15}H_{10}O_6$	286.0477	287.0548	-0.6	12.17	876007	+H	153.0178[M+H-$C_8H_5O_2$]
45	Luteolin 5-glucoside	$C_{21}H_{20}O_{11}$	448.1006	449.1088	2.1	12.17	241475	+H	443.1129[M+H-OH]/287.0548[M+H-Glc]/153.0178[M+H-Glc-$C_8H_5O_2$]
46	6-hydroxyapigenin	$C_{15}H_{10}O_6$	286.0477	287.0547	-0.9	12.52	338578	+H	193.0126[M+H-C_6H_5O]/147.0437[M+H-$C_6H_3O_4$]
47	Anhydrosafflor yellow B	$C_{48}H_{52}O_{26}$	1044.2747	1045.2801	-1.7	12.53	3589900	+H	883.2274[M+H-Glc]/721.1749[M+H-2Glc]
48	Kaempferol-3-O-D-galactoside	$C_{21}H_{20}O_{11}$	448.1006	471.0891	-1.5	12.6	16213	+Na	271.0587[M+H-OGlc]
49	6-methoxykaempferol	$C_{16}H_{12}O_7$	316.0583	317.0652	-1.1	12.78	503109	+H	302.0415[M+H-CH_3]/287.0543[M+H-CH_3-OH]
50	6-(Aminomethyl)-3-methyl-flavone hydrochloride	$C_{17}H_{16}ClNO_2$	301.0870	302.0515	4.2	12.78	5042	+H	173.5222[M+H-C_6H_5-NH_3]/121.0277[M+H-C_9H_8-CH_5N]
51	Baicalin	$C_{21}H_{18}O_{11}$	446.0849	447.0923	0.3	13.08	73419	+H	271.0593[M+H-$C_6H_8O_6$]/169.0124[M+H-$C_6H_8O_6$-C_7H_6]
52	Dihydrochalcone	$C_{15}H_{14}O$	210.1045	211.1201	0.7	14.66	17149	+H	91.0539[M+H-C_8H_7O]/77.0382[M+H-C_9H_9O]
53	Saffloquinoside E	$C_{30}H_{34}O_{15}$	634.1898	657.1799	1.3	15.21	6176	+Na	529.1637[M+H-OH-CH_3O-$C_2H_3O_2$]/471.1249[M+H-Glc]/371.0652[M+H-Glc-C_6H_5O-$C_2H_3O_2$]

54	Kaempferol3-O-caffeoyl-sophoroside7-O-glucoside	$C_{42}H_{46}O_{24}$	934.2379	957.2270	-0.2	16.71	873179	+Na	667.2118[M+H-$C_{27}H_{39}O_{19}$]/569.1070[M+H-$C_{24}H_{25}O_{16}$]449.0500[M+H-$C_{23}H_{13}O_{10}$]289.0700[M+H-$C_{15}H_{13}O_6$]
55	Luteolin	$C_{15}H_{10}O_6$	286.0477	287.0544	-2	16.75	90094	+H	153.0176[M+H-$C_8H_6O_2$]
56	Carthamin	$C_{43}H_{42}O_{22}$	910.2168	933.2025	-3.8	16.75	30531	+Na	569.1070[M+H-2Glc-OH]/437.7508[M+H-2Glc-C_8H_7O-OH]
57	N1,N5,N10-(Z)-tri-p-cou-maroylspermidine	$C_{34}H_{37}N_3O_6$	583.2682	584.2753	-0.4	18.44	2740807	+H	438.2391[M+H-$C_{25}H_{32}N_3O_4$]/420.2283[M+H-$C_{25}H_{30}N_3O_3$]/275.1754[M+H-$C_{16}H_{23}N_2O_2$]/147.0437[M+H-$C_9H_7O_2$]
58	Apigenin	$C_{15}H_{10}O_5$	270.0528	271.0598	-0.9	18.63	175455	+H	119.0488[M+H-$C_7H_4O_4$]/153.0779[M+H-C_8H_6O]
59	Tinctormine	$C_9H_{12}N_6O_4S$	300.0641	301.0705	-2.8	19.09	54821	+H	286.4679[M+H-CH_3]/168.0046[M+H-$C_4H_6O_3N$-NH_2]/140.0989[M+H-$C_4H_6O_3N$-CH_2ON]
60	Palmitic acid	$C_{16}H_{32}O_2$	256.2402	257.2737	-1.4	22.63	33846	+H	201.0458[M+H-$C_3H_5O_2$]
61	9,11-Octadecadienoic acid,13-oxo-,(9Z,11E)-	$C_{18}H_{30}O_3$	294.2195	295.2264	-1.3	22.67	36974	+H	277.2147[M+H-H_2O]

Tabel 2. Identifying the components in ESI$^-$ mode

NO.	Component name	Formula	Theoretical (m/z)	Observed (m/z)	Mass error (ppm)	tR (min)	Response	Adducts	Ion fragment
1	N-feruloyltryptamine	$C_{20}H_{20}N_2O_3$	336.1474	381.1185	4.6	0.6	9653	+HCOO	191.0555[M-H-$C_{10}H_{10}N$]/179.0552[M-H-$C_{10}H_{10}N$-CH_3]/161.0450[M-H-$C_{10}H_{10}N$-OCH_3]

2	Saffloquinoside D	$C_{27}H_{34}O_{18}$	646.1745	645.1671	-0.2	1.24	5154	-H	447.0737[M-H-Glc]/285.0396[M-H-2Glc]/243.0292[M-H-C_3H_2O-2Glc]/165.9898[M-H-2Glc-C_8H_8O]
3	D-Phenylalanine	$C_9H_{11}NO_2$	165.0790	164.0711	-3.4	2.38	61110	-H	147.0445[M-H-NH_2]/103.0549[M-H-NH_3-CHO_2]
4	Quercetin-3,7-O-β-D-glucopyranoside	$C_{27}H_{30}O_{17}$	626.1483	625.1411	0.2	3.06	90228	-H	565.1206[M-H-$C_2H_4O_2$]/417.0827[M-H-Glc-CH_2O]
5	Esculetin	$C_9H_6O_4$	178.0266	223.0241	-3.3	3.56	6240	+HCOO	153.0188[M-H-C_2H_2]/125.0236[M-H-C_3H_2O]
6	Cartormin	$C_{27}H_{29}NO_{13}$	575.1639	574.1521	-0.2	3.81	75518	-H	353.0873[M-H-GLC-$C_2H_4O_2$]/123.0083[M-H-C_6H_5O-$C_9H_9O_4N$-Glc]
7	Saffloquinoside C	$C_{27}H_{32}O_{17}$	628.1640	627.1571	0.7	4.56	547978	-H	609.1458[M-H-H_2O]/419.0981[M-H-RhaO-CH2O-OH]/299.0555[M-H-RhaO-OGlc]/205.0136[M-H-ORha-OGlc-C_6H_5O]
8	Chlorogenic acid	$C_{16}H_{18}O_9$	354.0951	353.0879	0.2	5.06	207654	-H	191.5549[M-H-$C_9H_7O_3$]/135.0447[M-H-$C_8H_8O_7$]
9	Saffloquinoside E	$C_{30}H_{34}O_{15}$	634.1898	679.1884	0.6	5.06	14462	+HCOO	566.1386[M-H-2OH-CH_2O]/375.0694[M-H-Glc-C_6H_5O]
10	6-Hydroxykaempferol-3,6,7-triglucoside	$C_{33}H_{40}O_{22}$	788.2011	787.1941	0.3	5.09	1060464	-H	625.1415[M-H-Glc]/463.0883[M-H-2Glc]
11	Hydroxysafflower yellow A	$C_{27}H_{32}O_{18}$	644.1589	643.1504	-1.8	5.24	20327	-H	625.1400[M-H-H_2O]/283.2329[M-H-2OGlc]
12	Syringin	$C_{17}H_{24}O_9$	372.1420	417.1399	-0.8	5.26	63484	+HCOO	189.0544[M-H-Glc-OH]/145.2916[M-H-Glc-OCH_3-CH_2O]
13	Roseoside	$C_{19}H_{30}O_8$	386.1941	431.1921	-0.5	6.58	276186	+HCOO	205.1224[M-H-OGlc]/189.0809[M-H-

									OGlc-OH]/165.0921[M-H-Glc-C_2H_4O-OH]
14	Yellow pigment A	$C_{26}H_{28}O_{16}$	596.1377	641.1362	0.4	6.88	40053	+HCOO	431.0974[M-H-Rha]/287.0551[M-H-Rha-Glc]
15	Cartorimine	$C_{15}H_{14}O_6$	290.0790	335.0769	-1.1	6.95	17349	+HCOO	255.0652[M-H-2OH]/143.0321[M-H-C_3H_2O-C_6H_5O]/135.0446[M-H-CHO_2-OH-C_6H_5O]
16	Saffloquinoside A	$C_{27}H_{30}O_{15}$	594.1585	639.1560	-1	7.28	62056	+HCOO	449.1086[M-H-Rha-CH_2]/287.0552[M-H-Glc-Rha-CH_2]
17	6-hydroxykaempferol-3,6,7-O-β-D-glucoside	$C_{34}H_{42}O_{21}$	786.2219	785.2136	-1.3	7.41	21628	-H	623.1259[M-H-Glc]/447.0948[M-H-O-2Glc]/300.2636[M-H-3Glc]
18	5,6,7,4'-tetrahydroxyisoflavone-6,7-di-O-β-D-glucopyranosid	$C_{27}H_{32}O_{16}$	612.1690	611.1618	0	7.66	2701286	-H	286.0478[M-H-2Glc]/119.0500[M-H-2OGlc-C_7HO_3]
19	kaempferol-3-O-β-rutinoside	$C_{27}H_{30}O_{15}$	594.1585	593.1512	0.1	7.74	124561	-H	365.0664[M-H-ORha-3OH]/192.0052[M-H-O2Rha-C_6H_5O]
20	Hexacosanoic acid	$C_{26}H_{52}O_2$	396.3967	441.3948	-0.3	7.84	23594	+HCOO	85.0287[M-H-$C_{22}H_{45}$]
21	6-hydroxykaempferol-6,7-O-β-D-glucoside	$C_{27}H_{30}O_{17}$	626.1483	625.1411	0.1	8.1	66919	-H	463.0880[M-H-Rha]/301.0350[M-H-Glc-Rha]
22	6-Hydroxykaempferol 3-Rutinoside-6-glucoside	$C_{33}H_{40}O_{21}$	772.2062	771.1992	0.3	8.24	1632678	-H	625.1407[M-H-Glc]/463.0873[M-H-Glc-Rha]/301.0346[M-H-2Glc-Rha]/271.0242[M-H-ORha-Glc-$C_6H_2O_3$-OH]

23	6-hydroxyapigenin-3,6-2-O-β-D-Glucoside	$C_{27}H_{30}O_{17}$	626.1483	625.1406	-0.7	8.29	1960279	-H	609.1460[M-OH]/463.0873[M-H-Glc]/301.0346[M-H-2Glc]
24	6-hydroxyapigenin-6-O-β-D-glucoside-7-O-β-D-glucuronide	$C_{27}H_{28}O_{17}$	624.1327	623.1252	-0.3	8.4	597063	-H	447.0929[M-H-$C_6H_9O_6$]/285.0398[M-H-Glc-$C_6H_9O_6$]/136.9874[M-H-$C_6H_9O_6$-Glc-$C_9H_6O_2$]
25	Carthamone	$C_{21}H_{20}O_{11}$	448.1006	447.0930	-0.7	8.4	64935	-H	285.0398[M-H-Glc]/136.9874[M-H-Glc-$C_7H_7O_2$]
26	6-hydroxyapigenin	$C_{15}H_{10}O_6$	286.0477	285.0396	-3.2	8.4	10447	-H	227.0290[M-H-OH]/165.9897[-H-C_8H_6O]/117.0338[M-H-$C_7H_4O_5$]
27	Saffomin C	$C_{30}H_{30}O_{15}$	630.1585	629.1507	-0.8	8.42	45465	-H	562.1550[M-H-4OH]/447.0929[M-H-$C_9H_8O_3$]/284.0318[M-H-Glc-$C_9H_8O_3$]
28	Safflower yellow A	$C_{27}H_{30}O_{16}$	610.1534	609.1459	-0.3	8.47	140923	-H	381.6110[M-H-OGlc-CH_2O-OH]/283.0244[M-H-2Glc]/187.3904[M-H-2Glc-$C_4H_2O_3$]
29	Kaempferol-3-O-β-D-xyloside	$C_{20}H_{18}O_{10}$	418.0900	463.1037	-0.4	9.05	201973	+HCOO	253.0491[M-H-OGlc-OH]/145.0286[M-H-OGlc-$C_6H_4O_3$]
30	Kaempferol-3-O-sophoroside	$C_{27}H_{30}O_{16}$	610.1534	609.1458	-0.5	9.35	1378148	-H	301.0347[M-H-Glc-C_6H_5O-2OH]/271.0243[M-H-OGlc-$C_6H_4O_3$-CH_2O]
31	6-hydroxykaempferol-7-O-glucoside	$C_{21}H_{20}O_{12}$	464.0955	463.0877	-1	9.47	1767272	-H	301.0344[M-H-Glc]
32	6-Hydroxykaempferol	$C_{15}H_{10}O_7$	302.0427	301.0343	-3.6	9.47	23703	-H	284.0320[M-H-OH]/139.0283[M-H-$C_9H_6O_3$]

33	Scutellarin	$C_{21}H_{18}O_{12}$	462.0798	461.0730	0.9	10.83	27085	-H	429.0828[M-H-2OH]/284.0324[M-H-$C_6H_6O_3$]
34	Kaempferol 3-O-β-D-allopyranoside	$C_{21}H_{20}O_{11}$	448.1006	447.0932	-0.1	11.2	86927	-H	271.0238[M-H-$C_6H_4O_3$-CH_3O-OH]/179.0339[M-H-$C_6H_4O_2$-Glc]
35	6-hydroxykaempferol-3,6-O-β-D-diglucoside	$C_{22}H_{22}O_{11}$	462.1162	507.1134	-2	11.95	55940	+HCOO	301.0342[M-H-Glc]/133.0283[M-H-Glc-$C_7H_4O_5$]
36	Safflower yellow B	$C_{48}H_{54}O_{27}$	1062.2853	1061.2779	0	12.18	313549	-H	611.1602[M-H-Glc-$C_{15}H_{10}O_6$-OH]/449.1870[M-H-Glc-$C_{21}H_{15}O_{11}$]/285.0396[M-H-Glc-$C_{15}H_{10}O_6$-Glc]
37	Luteolin 5-glucoside	$C_{21}H_{20}O_{11}$	448.1006	447.0932	-0.1	12.53	237525	-H	431.0964[M-OH]/286.0478[M-H-Glc]
38	Quercetin-7-O-β-D-glucoside	$C_{21}H_{20}O_{12}$	464.0955	463.0885	0.6	12.67	35398	-H	284.0321[M-H-Glc-OH]/146.9652[M-H-Glc-$C_8H_6O_3$]
39	Matairesinol-monoglucoside	$C_{26}H_{32}O_{11}$	520.1945	565.2085	0.3	13.06	15138	+HCOO	324.0996[M-H-Glc-CH_2O]/281.0805[M-H-Glc-$C_2H_2O_2$-CH_3]
40	Baicalin	$C_{21}H_{18}O_{11}$	446.0849	445.0775	-0.4	13.08	30948	-H	269.0449[M-H-$C_6H_8O_6$]
41	Crocin	$C_{44}H_{64}O_{24}$	976.3788	975.3743	-4.6	14.17	23129	-H	615.1684[M-H-2Glc]/487.1794[M-H-$C_7H_{10}O_2$-2Glc]
42	Kaempferol	$C_{15}H_{10}O_6$	286.0477	285.0400	-1.7	14.94	88959	-H	267.0288[M-H-H_2O]
43	Carthamin	$C_{43}H_{42}O_{22}$	910.2168	955.2161	1.2	16.71	2550120	+HCOO	461.1085[M+HCOO-GLC-$C_{15}H_7O_6$]/450.1124[M+HCOO-Glc-$C_{16}H_9O_6$]
44	Luteolin	$C_{15}H_{10}O_6$	286.0477	285.0400	-1.7	16.76	73610	-H	132.0210[M-H-$C_7H_4O_4$]
45	Naringenin	$C_{15}H_{12}O_5$	272.0685	271.0605	-2.4	18.05	8048	-H	145.0289[M-H-$C_6H_4O_3$]/132.0570[M-H-$C_6H_4O_3$-OH]/93.0337[M-H-$C_9H_4O_4$]

46	N1,N5,N10-(Z)-tri-p-coumaroylspermidine	$C_{34}H_{37}N_3O_6$	583.2682	582.2612	0.4	18.44	869253	-H	462.2030[M-H-C_8H_7O]/316.1664[M-H-$C_9H_8O_2$-C_8H_7O]
47	Apigenin	$C_{15}H_{10}O_5$	270.0528	269.0450	-2.2	18.64	70120	-H	117.0339[M-H-$C_7H_4O_4$]
48	6-hydroxyapigenin	$C_{15}H_{10}O_6$	286.0477	285.0398	-2.2	18.86	89907	-H	166.9951[M-H-C_8H_6O]/145.286[M-H-$C_6H_4O_4$]
49	Tinctormine	$C_9H_{12}N_6O_4S$	300.0641	299.0556	-4.1	19.09	23830	-H	271.0602[M-H-NH_2-N]/183.4349[M-H-CH_2ON-C_2H_4ON]
50	6-methoxykaempferol	$C_{16}H_{12}O_7$	316.0583	315.0506	-1.4	19.19	31599	-H	169.0132[M-H-C_6H_4-4OH]
51	Myristic acid	$C_{14}H_{28}O_2$	228.2089	273.2221	-2.3	21.32	20045	+HCOO	255.2068[M-H-OH]/227.1967[M-H-CHO_2]
