# Supplementary material for: Mechanism Repositioning Based on Integrative Pharmacology: Anti-Inflammatory Effect of Safflower in Myocardial Ischemia–Reperfusion Injury
Source: Int J Mol Sci. 2023 Mar 10;24(6):5313. doi: 10.3390/ijms24065313 (PMC10048972; doi:10.3390/ijms24065313)
Supplement: Supplementary file 1 [file ijms-24-05313-s001.zip › supplementary file S10.pdf]

Protein	components	binding affinities
PRKCA	rutin	-8.1
	6_Hydroxykaempferol 3_Rutinoside_6_glucoside	-11.2
	6_Hydroxykaempferol	-9.1
	Apigenin	-9.1
	Baicalin	-9.9
	Eriodictyol	-9.4
	Hydroxysafflor yellow A	-7.8
	Kaempferol	-9.2
	Luteolin	-9.5
	quercetin	-9.7
	Rutin	-8.1
PIK3CG	Eriodictyol	-8.0
	Hydroxysafflor yellow A.	-5.9
	Kaempferol	-7.6
	Luteolin	-7.8
	quercetin	-7.7
	Rutin	-9.0
AKT1	Eriodictyol	-8.3
	Hydroxysafflor yellow A.	-7.7
	Kaempferol	-7.5
	Luteolin	-8.4
	quercetin	-8.1
	Rutin	-9.2
