# Supplementary material for: Mechanism Repositioning Based on Integrative Pharmacology: Anti-Inflammatory Effect of Safflower in Myocardial Ischemia–Reperfusion Injury
Source: Int J Mol Sci. 2023 Mar 10;24(6):5313. doi: 10.3390/ijms24065313 (PMC10048972; doi:10.3390/ijms24065313)
Supplement: Supplementary file 1 [file ijms-24-05313-s001.zip › supplementary file S11.pdf]

The mRNA levels of PI3K and HIF1α

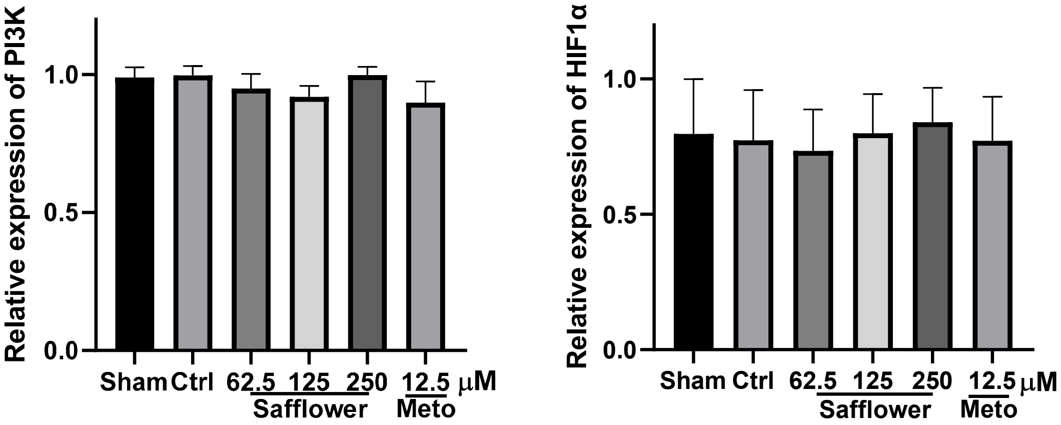

Supplementary figure S1. The expression of mRNA levels of PI3K and HIF1α
Values are means ± SEM, $^{\#}P < 0.05$, $^{\#\#}P < 0.01$, $^{\#\#\#}P < 0.001$ vs. sham, respectively; $^{*}P < 0.05$, $^{**}P < 0.01$, $^{***}P < 0.001$ vs. Control, respectively; One-way ANOVA.
